# Public Awareness and Practice Regarding Over-the-Counter Medications: A Cross-Sectional Study in Al-Ahsa, Saudi Arabia

**DOI:** 10.7759/cureus.58410

**Published:** 2024-04-16

**Authors:** Sahbanathul Missiriya Jalal, Suhail Hassan Jalal

**Affiliations:** 1 Department of Nursing, College of Applied Medical Sciences, King Faisal University, Al-Ahsa, SAU; 2 Department of Pharmacy, Jaya College of Pharmacy, The Tamil Nadu Dr. MGR Medical University, Chennai, IND

**Keywords:** knowledge, practice, public awareness, self-medication, otc, over-the-counter drugs

## Abstract

Background

Pharmaceuticals classified as over-the-counter (OTC) medications are also known as self-medications, in which drugs are sold directly to customers without a valid prescription. According to the World Health Organization, self-medication refers to taking medication for ailments that one has self-diagnosed. The public viewed OTC medications as safer, more effective, and beneficial, but misuse can lead to other health issues. Therefore, this study aimed to assess awareness and practices regarding OTC medications.

Methodology

The study employed a cross-sectional design involving the public residing in Al-Ahsa in eastern Saudi Arabia. The study setting was primary health centers (PHCs) in Al-Ahsa. Four PHCs were chosen by the cluster sampling method. One PHC from each of the four health clusters (northern, southern, middle, and eastern) was selected and a total of 326 people were chosen by simple random sampling from those four PHCs. Data were collected through a structured self-administered questionnaire. Descriptive and inferential statistics such as mean, standard deviation, chi-square, and correlation analyses were used to analyze the results.

Results

The mean age of the participants was 38.26 ± 9.73 years. The overall mean knowledge score regarding OTC medications was 14.21 (SD = 3.3). About 39 (11.96%) of the participants had adequate knowledge, 184 (56.44%) had moderately adequate knowledge, and 103 (31.6%) had inadequate knowledge about the safety of using OTC medications. The overall mean score of practices was 20.7 ± 4.42. The chi-square test results showed a significant (p < 0.01) association between the level of knowledge and age, occupation, nationality, and marital status. Additionally, a positive linear relationship (r=+.386) was found between knowledge and practices regarding OTC medications.

Conclusion

In conclusion, many people in the present study had moderate knowledge and good practices regarding OTC medications. To protect the public from harm, there is an urgent need for more concrete regulatory control over OTC drugs and self-medication. So, it is recommended to create awareness about the proper use of OTC medications.

## Introduction

Self-medication (SM) is the practice of taking medication for symptoms that one has self-diagnosed without a legitimate prescription. The substances most widely used in SM are over-the-counter (OTC) drugs, which are used to treat common health issues [1]. According to the World Health Organization (WHO), SM is the use of medications to address illnesses or symptoms that one has self-diagnosed, as well as the sporadic or continuous use of prescription medications for long-term or recurrent illnesses or symptoms even after the recommended time has passed [2].

Recklessly administered SM was often seen as superfluous, but today it is recognized as a crucial component of self-care. OTC medications can be sold to customers without a valid prescription from a pharmacy. OTC medications address a wide range of illness-related symptoms, such as pain, fungal infections, diarrhea, heartburn, constipation, coughing, and colds. These medications are typically found online, at pharmacies, grocery stores, and gas stations on shelves [3].

The practice of OTC drugs is spreading around the globe. Based on the target audience and country, studies show that the global prevalence of SM varies between 11.2% and 93.7%. This indicates that a large proportion of the world’s population uses OTC drugs without medical consulting [4]. Globally, the rising prevalence of improper SM is raising concerns about public health, especially regarding the development of antibiotic resistance brought on by frequent and incorrect use of antibiotics [5].

The prevalence of OTC drugs is common in Saudi Arabia. According to reports, 81.4% of the general population in Saudi Arabia has used pharmaceuticals without a prescription at some point in their lives. Analgesics were the most self-administered drugs, followed by antipyretics, cough syrups, eye drops, antibiotics, flu medication, heartburn medication, medicines for joint pain, and so on [6].

Poor understanding of OTC drugs can have a direct negative impact on health, including excessive use or non-adherence to treatment plans [7]. Since social media has been more widely available than healthcare, the public has become confused and afraid due to false information about medications, disease etiology, treatments, and prevention [8]. Study findings in the Dammam district revealed that more than half of the people (66%) lacked appropriate understanding regarding OTC pharmaceuticals. Purchases of OTC medications from community pharmacies were rather popular in the Qassim region (75%), with the most frequent reason for doing so being the renewal of a prior prescription from a medical professional (30%) [9].

The practice of inappropriate SM carries several possible dangers and bad outcomes to human health, including the risk of toxicity or overuse, adverse drug reactions, drug duplication, incorrect drug use, missing diagnosis, drug dependence, drug-drug, drug-food, and drug-disease interactions, and the risk of drug dependence [10]. Some studies showed that people rarely or never check the expiry date of the OTC medications they took. This is concerning since failing to verify a medication's expiration date might result in an accumulation of expired pharmaceuticals in the home and numerous negative side effects [11].

SM may create damage at both individual and community levels. When people take medications on their own without consulting a doctor, they run the risk of misdiagnosing and treating themselves, failing to get to the right medical facility, taking the medication at the wrong time, taking it insufficiently, experiencing side effects, developing drug dependence, microbial resistance, and wasting national resources [12].

The public views OTC medications as easier to get, safer, effective, and having many more benefits. The misuse and inappropriate use of OTC medications may lead to many health issues. Knowledge and practice are the most used methods to explain how an individual’s knowledge and beliefs influence changes in health behavior. Therefore, this study aimed to assess their awareness and practice regarding OTC medications.

## Materials and methods

Study design

This study adopted a cross-sectional approach to assess the level of knowledge and practice of people regarding OTC. Ethical approval was obtained from the Ethics Committee at the Deanship of Scientific Research, King Faisal University (approval number: 969/2023). Before their participation, all participants signed an informed consent form in either Arabic or English. Throughout the study, ethical considerations and confidentiality of participants’ information were strictly maintained using an anonymized data collection tool comprising various variables to assess knowledge and practices about OTC medications among people.

Study setting and participants

The present study was conducted in the selected primary health center (PHC), in Al-Ahsa from the eastern region of the Kingdom of Saudi Arabia. For covering the Al-Ahsa population, 68 PHCs were functioning. Four PHCs were chosen by cluster sampling method from them. One PHC from each of the four health clusters (northern, southern, middle, and eastern), and the participants were chosen by simple random sampling from those four PHCs. The people aged 18 years and above including both genders residing in Al-Ahsa and showed their willingness to participate in the survey were included. People from the healthcare field were excluded from the study.

Study sampling

The sample size was estimated considering the single population proportion formula using Epi‑Info™ software, version 7.2. According to the Al-Ahsa Governorate estimation in the year 2023, around 1.3 million people were living. Assuming 50% of the study population possessed adequate knowledge and good practice from the previous study [13] with a 5% acceptable margin of error at a 95% confidence level, accounting for the ﬁnite population of 385 was calculated. After exclusion, 326 participants were selected by a simple randomization method, using computer-generated random numbers. The formula used for sample calculation was n = [DEFF*Np(1-p)]/ [(d2/Z21-α/2*(N-1)+p*(1-p)].

Data collection

A structured questionnaire was constructed by the authors and used to collect the data. This questionnaire is an original tool, prepared in English and translated into Arabic, and was evaluated by a panel of experts (three physicians, two pharmacists, and two nursing professionals) to validate the tool. According to their constructive suggestions, the tool was modified. The structured questionnaires consisted of three parts: 1) demographic variables, 2) knowledge questionnaire, and 3) practice questionnaire. A pilot study was conducted to improve the tool. In the pilot, the reliability of the knowledge questionnaire was tested by the test and retest method for each item, internal consistency for each construct (r = 0.876), and the reliability of the practice questionnaire (r = 0.901) using Cronbach’s alpha coefficient. The time duration to fill in the questionnaire ranged from 15 to 20 minutes. Information was included in the tool with an introduction, objectives of the study, and ensuring privacy and confidentiality before distribution. Participants were informed that their participation in the research was voluntary without any financial support. Informed consent was obtained from all the participants before the data collection.

Demographic variables

The demographic variables included age, gender, educational level, occupation, nationality, marital status, and source of health information.

Knowledge questionnaire

The second part of the tools included 25 multiple-choice questions regarding the OTC medications. Each question has one most appropriate correct option and 3 wrong options. For the correct answer to each question “1” point was provided, while every incorrect answer counted as “0”. An overall score of ≥19 (more than 75%) was considered adequate knowledge, the score range between 13 and 18 (50% - 75%) was considered moderately adequate knowledge, and a score ≤12 (less than 50%) was scored inadequate knowledge.

Practice questionnaire

The third part of the tools is about the practice of people regarding OTC medications. It consisted of 12 questions with many different options such as “Never”, “Sometimes” and “Always” and a point of “1”, “2” and “3” was given respectively. The total score ranged between 1 and 17 (1% - 50%) considered as poor practice, the score between 18 and 27 (51% -74%) considered to be good practice, and the range between 28 and 36 (75% - 100%) demonstrated as best practice. 

Data analysis

Statistical analysis was performed with IBM SPSS Statistics for Windows, Version 21 (Released 2012; IBM Corp., Armonk, New York, United States). The statistical significance level was set at p < 0.05. Descriptive statistics, such as frequency and percentages, were used for categorical variables, and the mean and standard deviation (SD) were used for continuous variables. The relationship between subjects' demographic characteristics and knowledge level was assessed using chi-squared tests. Pearson’s correlation test was used to relate the level of knowledge and practice of the participants.

## Results

Demographic variables 

The demographic characteristics of the study participants were analyzed and are shown in Table 1. A total of 326 people were included in the analysis, of which 144 (44.2%) were in the age range of 31-40 years. The mean age of the people was 38.26 (SD ± 9.73) years. Approximately, 182 (55.8%) women participated in the study. In terms of education, half of the people 163 (50%) had a high school level of education, and 75 (23%) studied up to graduate level. Regarding their occupation, most of them 262 (80.4%) were employed. Among them, 275 (84.4%) were Saudis while the remaining 33 (15.6%) were non-Saudis married. The majority of people 179 (84.4%) were Saudis while the remaining 51 (15.6%) were non-Saudis. Among them, 265 (81.3%) were married. Regarding the source of obtaining health information, mostly, 190 (58.3%) received from social media, with only 33 (10.1%) receiving from healthcare professionals.

**Table 1:** Demographic characteristics of the participants (n = 326) N: Number; %: Percentage

Variables	Category	Number (N)	Percentage (%)
Age (years)	18-30 years	67	20.6
	31-40 years	144	44.2
	41-50 years	72	22.1
	More than 50 years	43	13.2
Gender	Male	144	44.2
	Female	182	55.8
Education	Primary level	42	12.9
	High school level	163	50.0
	Graduate level	75	23.0
	Post-graduate level	20	6.1
	Others (diploma)	26	8.0
Occupation	Employed	262	80.4
	Unemployed	64	19.6
Nationality	Saudi	275	84.4
	Non-Sadi	51	15.6
Marital Status	Unmarried	28	8.6
	Married	265	81.3
	Others	33	10.1
Source of Health Information	Social Media	190	58.3
	Family Members & Relatives	54	16.6
	Friends& Others	49	15.0
	Health Professionals	33	10.1

Knowledge regarding OTC medications

The overall mean score of knowledge regarding OTC medications was 14.21 (SD = 3.3) as shown in Table 2. About 39 (11.96%) of the participants had adequate knowledge, 184 (56.44%) had moderately adequate knowledge, and 103 (31.6%) had inadequate knowledge about the safety of the OTC medications. The mean scores with SD of adequate, moderate, and inadequate knowledge were 19.41 ± 0.64, 15.29 ± 1.61, and 10.3 ± 1.43 respectively.

**Table 2:** Knowledge of people regarding OTC medications (n = 326) N: Number; %: Percentage, SD: Standard deviation

Level of Knowledge	N (%)	Knowledge Score		Skewness	Kurtosis
		Mean	SD		
Adequate Knowledge (Score between 19 and 25)	39 (11.96)	19.41	0.64	1.319	0.69
Moderate Knowledge (Score between 13 and 18)	184 (56.44)	15.29	1.61	0.23	-1.054
Inadequate Knowledge (Score between 0 and 12)	103 (31.6)	10.3	1.43	-0.652	-0.183
Overall	326 (100)	14.21	3.3	-0.091	-0.777

Practice regarding OTC medications

Regarding the practice of OTC medications (Table 3), among 326 participants, only 31 (9.5%) used to take OTC for minor health issues. Around 167 (51.2%) never discard OTC medications, even if they find changes in the shape, color, or odor of the medications. In addition, 43 (13.2%) people used to store OTC drugs in a properly sealed medicine box. Approximately 121 (37.1%) took antibiotics without consulting physicians. Around 80 (24.5%) used anti-allergic drugs for sleeping purposes. Most of them, 213 (65.3%), never recognize or bother about the adverse reactions of OTC medications. Regarding the drug-to-drug reactions, 124 (38%) people understood sometimes. Only 47 (14.4%) people thought that OTC medications could be advised when necessary. Among them, 68 (20.9%) used to take OTC medications with precautions and safety in mind. While taking OTC medications, 80 (24.5%) never verify the expiration date. Very few people (3.1%) followed the OTC medications by reading the leaflets for indications and contraindications. One hundred and forty-eight participants (45.4) used to buy OTC medication after consulting pharmacists. The overall mean score of practice regarding OTC medications was 20.7 ± 4.42, as shown in Table 4. The mean scores for best, good, and poor practice were 28.75 ± 0.84, 21.48 ± 2.52, and 15.41 ± 1.51, respectively.

**Table 3:** Frequency distribution of practice regarding OTC medications (n = 326) N: Number; %: Percentage OTC: Over-the-counter

Practice-related questions	Never		Sometimes		Always	
	N	(%)	N	(%)	N	(%)
Take OTC medications for minor health issues.	216	66.3	79	24.2	31	9.5
Discard OTC medications if there are changes in shape, or color, or odor.	167	51.2	106	32.5	53	16.3
Store OTC drugs only in the covered medicine box.	102	31.3	181	55.5	43	13.2
Take antibiotics only after the consultation of doctors.	121	37.1	137	42.0	66	20.2
Avoid antiallergic OTC drugs for sleeping.	80	24.5	172	52.8	71	21.8
Able to recognize and bother about the adverse reactions of OTC medications.	213	65.3	73	22.4	40	12.3
Understand the drug-to-drug reactions due to multiple OTC medications at a time.	148	45.4	124	38.0	54	16.6
OTC medications can be advised when necessary.	100	30.7	179	54.9	47	14.4
Take OTC medications with any precautions and safety.	120	36.8	136	41.7	68	20.9
Take OTC medications after verifying the expiry date.	80	24.5	176	54.0	67	20.6
Follow the OTC medications by reading the leaflet for indications and contraindications.	225	69.0	91	27.9	10	3.1
Buy OTC medication after consulting the pharmacists.	106	32.5	148	45.4	72	22.1

**Table 4:** The descriptive statistics of people regarding the practices of OTC medications (n = 326) N: Number; %: Percentage, SD: Standard deviation OTC: Over-the-counter

Level of Practice	N (%)	Practice Score		Skewness	Kurtosis
		Mean	SD		
Best Practice (Score between 28 and 36)	36 (11.04)	28.75	0.84	0.82	-1.8
Good Practice (Score between 18 and 27)	205 (62.89)	21.48	2.52	0.414	-0.652
Poor Practice (Score between 1 and 17)	85 (26.07)	15.41	1.51	-0.733	-0.384
Overall Practice Score	326 (100)	20.7	4.42	0.288	-0.613

Association between knowledge and selected demographic variables

The association between knowledge and selected demographic variables is shown in Table 5. The chi-square test result proved that there was a significant (p < 0.01) association found between the level of knowledge and age, occupation, nationality, and marital status. Gender and education were not associated with the level of knowledge and showed non-significance.

**Table 5:** Association between knowledge and selected demographic variables (n=326) X2: Chi-Square; *: Significant at p < 0.05; NS: Non-significant

Variables	Category	Adequate	Moderate	Inadequate	Chi-Square Tests
Age (years)	18-30 years	5	8	54	X^2^ = 157.107 p = 0.001*
	31-40 years	6	99	39	
	41-50 years	8	57	7	
	More than 50 years	20	20	3	
Gender	Male	14	88	42	X^2^ = 2.561 p = 0.278 NS
	Female	25	96	61	
Education	Primary level	6	26	10	X^2^ = 11.252 p = 0.188 NS
	High school level	15	85	63	
	Graduate level	11	42	22	
	Post-graduate level	3	12	5	
	Others (diploma)	4	19	3	
Occupation	Employed	28	171	63	X^2^ = 44.307 p = 0.001*
	Unemployed	11	13	40	
Nationality	Saudi	35	143	97	X^2^ = 14.527 p = 0.001*
	Non-Sadi	4	41	6	
Marital Status	Unmarried	0	7	21	X^2^ = 29.751 p = 0.001*
	Married	32	160	73	
	Others	7	17	9	
Source of Health Information	Social media	22	109	59	X^2^ = 8.661 p = 0.194 NS
	Family Members & Relatives	7	28	19	
	Friends & Others	2	32	15	
	Health Professionals	8	15	10	

Correlation between knowledge and practice

**Figure 1:**
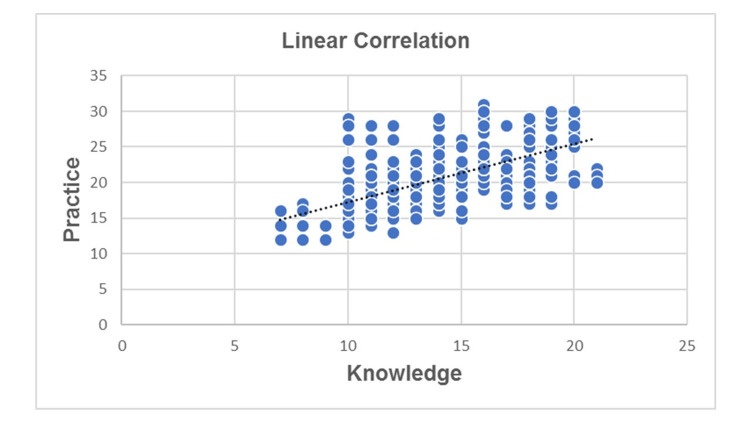
Linear correlation between knowledge and practice regarding OTC medications (n=326) r=+.386 OTC: Over-the-counter

The Pearson’s correlation test (Table 6) evidenced that there was a correlation between knowledge and practice regarding OTC medications at p <0.05. The positive linear relationship is depicted in Figure 1.

**Table 6:** Pearson correlation between knowledge and practice regarding OTC medications (n=326) a. Estimation is based on Fisher's r-to-z transformation. OTC: Over-the-counter

Variables	Pearson Correlation	Sig. (2-tailed)	95% Confidence Intervals (2-tailed)^a^	
			Lower	Upper
Knowledge Score - Practice Score	0.386	P = 0.001*	0.289	0.475

## Discussion

Our study evaluated the public’s knowledge and usage of OTC drugs and their safety. We discovered that the majority of the participants possessed information that was either insufficient or moderate. This finding was supported by a study conducted at primary healthcare centers in Palestine, in which 80% of the parents of children seeking medical attention were found to be ignorant [14]. According to the findings of a cross-sectional questionnaire study done in Northern Ireland, people's perceptions of the efficacy of OTC medications were based on their prior usage [15].

In our study, regarding the practice of OTC medication, very few participants (9.5%) used to take OTC medications for minor health issues. A survey was done to find out how common OTC medications are, what their indications are, and how much the general population knows about their negative effects. Half of them who were exclusive users of prescription non-steroidal anti-inflammatory drugs (NSAIDs) thought they were safer, compared to those who were exclusive OTC users; others thought OTC medications were safer. Of the exclusive OTC medication users, most did not know or thought they could experience NSAIDs' adverse effects [16].

Similarly, a descriptive study aimed to measure the frequency of analgesics’ self-use and assess the general population’s knowledge of their adverse effects. One important finding from this study was that most participants (70.4%) learned about one or more of these medications from a doctor. Despite this, their level of knowledge was not higher than that of participants who learned about these medications from other sources (p = 0.085) [17].

In the current study, half of the participants never throw away OTC medications, even if they notice changes in the pills' color, shape, or smell. Around 13.2% of the individuals also reported using a medicine box that should be properly sealed to store OTC medications. Roughly, 121 people (37.1%) used antibiotics without a doctor's advice. Approximately 24.5% of people utilized anti-allergy medications to fall asleep. The majority of them never notice or give a thought to OTC medicine side effects.

In Northwest Ethiopia, a similar study was conducted to evaluate medical and pharmacy students' knowledge, attitudes, and practices about the use of OTC drugs and associated factors, whereas the majority of them stated that they used to throw away OTC medications if they changed in form, color, or scent [18].

Regarding drug-to-drug responses, 38% of participants in our survey occasionally understood. Merely 14.4% of respondents believed that OTC drugs might be prescribed in some situations. Of them, 20.9% have previously taken over-the-counter drugs safely and with prudence. In a different investigation, 408 pertinent papers were examined for drug-to-drug interactions involving OTC medications that could have more detrimental effects [19].

Customers in Asmara pharmacy stores participated in a descriptive study, which revealed that 35% of respondents always read the package insert(s) and 73.9% always check the expiration date when buying over-the-counter medications [20]. Of the OTC drug users in our study, 24.5% never check the expiration date. Just 3.1% of individuals used OTC drugs as directed by reading the instructions for use and avoidance.

In terms of where people obtained their health information, half of the people got it from social media, while only 10.1% got it from medical experts in our study. This was compared with a study done to examine the applications and abuses of OTC painkillers in sub-Saharan Africa. The belief that an ailment is mild, difficulties accessing health services, and experience managing prior illnesses were the main causes of OTC medicine use. Family, friends, neighbors, pharmacies, and reading pamphlets handed out either in the neighborhood or at educational institutions were the sources of information about SM. OTC drug use was more frequently reported by females, individuals with less education than a secondary school diploma, and participants who were older than fifty [21]. Ghanaian pregnant women self-medicate were disproportionately high. In Ghana, actions might be taken to lower the high rate of SM during pregnancy to meet sustainable development goals related to maternal health [22]. A study conducted in Thailand's metropolitan areas revealed a significant prevalence of SM among individuals of working people. The study participants had various basic pharmaceutical knowledge errors, including misconceptions about antibiotic drug ideas [23]. 

An investigation was conducted to ascertain the frequency of SM and evaluate consumers' attitudes, knowledge, and perceptions of SM in Riyadh [24]. Overall, most participants had poor knowledge and negative perceptions regarding SM. More than 68% of participants did not know whether the medicine they bought was a prescription-only or OTC medication. The mean knowledge score was significantly associated with education level (p<0.001). In our study, controversially, knowledge level is not associated with education. However, the occupation was significantly associated with knowledge. The analysis revealed that those who are employed had a statistically significantly higher mean knowledge score than those who are unemployed.

During the COVID-19 epidemic in Brazil, a study examined the prevalence, profile, and associated factors of SM. The findings showed a clear link between OTC medications and SM, with paracetamol and dipyrone being the most commonly utilized analgesics [25]. In the aftermath of the conflict in northern Uganda, a study evaluated the frequency and correlates of antibiotic SM. It was shown that SM with antimicrobial medications for the treatment of illness symptoms is a prevalent practice, even in the face of knowledge of the risks involved [26]. A study was done to examine the frequency and related variables of SM with medications in Brazil. According to the survey, the majority of medications taken for SM were categorized as non-prescriptive [27].

In the current study, a positive correlation (r=+.386) was found between knowledge and practice regarding OTC medications (p <0.05). However, another study that looked at the degree and sufficiency of knowledge, attitude, and practice among Saudi Arabian citizens in Riyadh provided evidence, indicating that residents with good knowledge possess better attitudes (r = 0.142, p < 0.001) and follow the proper practice (r = 0.256, p < 0.001) toward SM, in which there was no strong positive link found between SM knowledge and practice [28].

The strength of the present study was the selection of samples randomly to reduce bias. However, the present study has certain limitations. The people were selected only from four PHCs, due to the particular geographic location, its generalizability is limited. The prevalence of OTC drugs and the reasons for people choosing OTC drugs were not included. Hence, future studies are needed to investigate those and identify the health issues associated with taking SM.

## Conclusions

It was determined that most of the individuals 184 (56.44%) had moderately adequate knowledge, and some 103 (31.6%) had inadequate knowledge about the appropriate and secure handling of OTC medications. As per the results of the study, it is suggested that further information be provided regarding the hazards associated with SM, OTC medications, adverse drug reactions, and drug-to-drug reactions, as well as their side effects and contraindications. Policies must provide adequate facilities for people to receive medical treatments. There is an urgent need for more robust regulatory control over OTC pharmaceuticals and SM to safeguard the public from health risks related to these products. The national level of study is required to investigate the public's knowledge and practice of OTC drugs. Ministry of Health emphasizes awareness programs in the mass media and social media in their languages to decrease SM without prescriptions.
